# True Specialism

**Published:** 1883-02

**Authors:** 


### TRUE SPECIALISM.

It was a wise answer that a distinguished English physician and a world-renowned specialist returned to his interrogator when asked if he did not "go in for diseases of the skin?" replied, "Yes, for diseases of the skin and all that it contains."

In this single sentence is comprised a vast amount of good sense and sound philosophy. It represents scientific specialism; a specialism that is worthy, honorable and desirable, and that stands in striking contrast to the so-called specialism of which we hear so much now-a-days—to that species of specialism manufactured at short notice to be placed upon the market for the purpose of meeting an existing demand and for the behoof of the individual only, who forms the connecting link between the representatives of legitimate medicine and that numerous class of avowed charlatans who openly ply their vocation and roam at will over the country, seeking whom they may devour.

If the sentiment embodied in the above quotation animated all medical men who prefer to restrict their attention to a single line of practice, we should hear less of "crusades against specialism," of "the relation between the specialist and the general practitioner," etc., and we should find a more cordial and more confiding intercourse springing up between the special and the general practitioners of the healing art. Because, on the one hand, that narrow routine which a limited knowledge necessitates would give place to more liberal and more reliable practice, and, on the other, the disreputable means commonly resorted to for proclaiming the self-assumed superiority in the treatment of certain diseases would be discountenanced to such a degree by the better class of specialists that a very potent, a really insurmountable, barrier to unrestrained association would be removed.

When specialists honestly place their claims to superior skill in the management of particular diseases upon a sufficiently broad basis and not only recognize the fact, but observe the rule, that they are subject to the same ethical requirements to which the profession at large subscribes, they will find that their critics will speedily grow less and their professional standing will be decidedly improved.

